# Acute interval running induces greater excess post-exercise oxygen consumption and lipid oxidation than isocaloric continuous running in men with obesity

**DOI:** 10.1038/s41598-024-59893-9

**Published:** 2024-04-22

**Authors:** Lang Jiang, Yihong Zhang, Zhengzhen Wang, Yan Wang

**Affiliations:** 1 School of Sports Medicine and Rehabilitation, Beijing Sport University, Xinxi Road, No 48, Beijing, China; 2 School of Public Service Management, Chongqing Vocational College of Transportation, Xiangfu Avenue, No 555, Chongqing, China; 3 School of Physical Education, Hunan University of Science and Engineering, Yangzitang Road, No 130, Yongzhou, Hunan China

**Keywords:** Physiology, Diseases, Health care, Medical research

## Abstract

Studies seem to show that high-intensity interval training (HIIT) is a more time-efficient protocol for weight loss, compared with moderate-intensity continuous training (MICT). Our aim was to compare the acute effects of energy expenditure (EE) matched HIIT vs. MICT on excess post-exercise oxygen consumption (EPOC) and substrate metabolism in male college students with obesity. Twenty-one untrained male college students (age, 22 ± 3 years; body fat, 28.4 ± 4.5%) completed two acute interventions (~ 300 kcal) on a treadmill in a randomized order: (1) HIIT: 3 min bouts at 90% of maximal oxygen uptake (VO_2max_) with 2 min of recovery at 25% of VO_2max_; (2) MICT: 60% of VO_2max_ continuous training. EPOC and substrate metabolism were measured by indirect calorimetry during and 30 min after exercise. Results showed that EPOC was higher after HIIT (66.20 ± 14.36 kcal) compared to MICT (53.91 ± 12.63 kcal, *p* = 0.045), especially in the first 10 min after exercise (HIIT: 45.91 ± 9.64 kcal and MICT: 34.39 ± 7.22 kcal, *p* = 0.041). Lipid oxidation rate was higher after HIIT (1.01 ± 0.43 mg/kg/min) compared to MICT (0.76 ± 0.46 mg/kg/min, *p* = 0.003). Moreover, the percentage of energy from lipid was higher after HIIT (37.94 ± 14.21%) compared to MICT (30.09 ± 13.54%, *p* = 0.020). We conclude that HIIT results in greater total EE and EPOC, as well as higher percentage of energy from lipid during EPOC than EE matched MICT in male college students with obesity.

## Introduction

Obesity has become a global issue in the field of public health, increasing the risk of various chronic diseases, such as type 2 diabetes mellitus and cardiovascular diseases^1^. Notably, the prevalence of obesity among university students is not optimistic^2^, which might be related to unhealthy lifestyle at a special stage of life^3^.

The essence of obesity is positive energy balance, which is comprehensively determined by genetic factors, dietary habits, physical activity, and social environment^4^. Lifestyle interventions, especially exercise interventions, play a critical role in weight management^5^.

High-intensity interval training (HIIT) enables subjects to achieve higher oxygen uptake and heart rate levels and improve body composition^6,7^ with shorter-time even lower-exercise volume commitment^8,9^ compared with moderate-intensity continuous training (MICT). Accordingly, HIIT is increasingly considered as a time-efficient type of exercise. Also, study has suggested that HIIT leads to more favorable effects in improving cardiorespiratory fitness and decreasing visceral adipose tissue, as well as generating greater energy expenditure (EE) during the excess post-exercise oxygen consumption (EPOC) period^10,11^.

EPOC is defined as the elevation in oxygen uptake and EE following exercise compared with the baseline period^12^, which is a key component contributing to the total EE. More importantly, the increase in lipid oxidation during EPOC period will be beneficial for fat loss. Long-term exercise interventions have shown that both HIIT and MICT could effectively reduce visceral and abdominal subcutaneous adipose tissue in young women with obesity, whereas HIIT is more superior^10^. Although some relative long-term intervention studies have demonstrated the benefits of HIIT for fat loss and general health, they have limitations. These studies paid less attention to substrate metabolism, especially lipid metabolism. Therefore, more researches concentrating on the effects of different exercise interventions on lipid metabolism during and after exercise are still needed.

Currently, more research evidence has the propensity to show that HIIT results in greater EPOC and lipid oxidation compared with MICT, with one explanation that greater EPOC is of relevance to the higher exercise intensity during HIIT^13–16^. Given that different study populations were recruited and a variety of exercise protocols with respect to exercise intensity and EE were performed during exercise, the evidence still remains controversial.

From the perspective of variable control, it is feasible to minimize the variables in exercise protocols, which could be more convincing to explain the results between interventions. In that respect, EE matched protocols could be applied to investigate relevant mechanisms.

Again, although beneficial metabolic effects on body composition produced by HIIT are seen^6,17^, further investigations are still warranted to explore the detailed substrate metabolism, especially lipid metabolism, during EPOC period. And if these studies combine with the actual EE required in weight loss, they would provide more insightful evidence for obesity practices.

Therefore, the aim of this study was to compare the effects of an acute session of EE matched (~ 300 kcal) interval- vs. continuous running on EPOC and substrate metabolism in male college students with obesity. It was hypothesized that HIIT would result in greater magnitude of EPOC and lipid oxidation than EE matched MICT.

## Methods

### Participants

Subjects were recruited from Beijing Sport University via online media and offline advertisements. Inclusion criteria were male, 18–25 years old, and body fat > 20%^18^. Exclusion criteria were cardiopulmonary or metabolic diseases, sports risk determined by the Physical Activity Readiness Questionnaire (PAR-Q), sports injuries limiting movement, and smoking. Thirty-one subjects expressed interest in this study. After screening, five subjects were ineligible. Two did not participate in the baseline test due to personal reasons, and three withdrew due to a cold. Thus, twenty-one male college students were analyzed for this cross-over study. This study was approved by the Ethical Committee for Human Experiments of Beijing Sport University, and the Declaration of Helsinki was followed by all the participant researchers. Written informed consent was obtained from each participant before the formal test. Baseline characteristics are presented in Table 1.

**Table 1:** Baseline characteristics.

Variables	Outcomes
Age (years)	22 ± 3
Height (cm)	178.5 ± 6.5
BM (kg)	88.6 ± 12.9
FFM (kg)	63.3 ± 6.5
BMI (kg/m^2^)	27.7 ± 3.1
Body fat (%)	28.3 ± 4.5
Resting HR (bpm)	78 ± 8
Resting VO2 (ml/kg/min)	3.5 ± 0.5
HRmax (rpm)	186 ± 14
VO2max/BM (ml/kg/min)	33.4 ± 5.1
VO2max/FFM (ml/kg/min)	46.4 ± 5.9

Results are presented as mean ± SD.

*BM* body mass, *FFM* fat free mass, *BMI* body mass index, *HR* heart rate, *VO*_*2*_ oxygen uptake, *HRmax* maximal heart rate, *VO*_*2max*_ maximal oxygen uptake.

### Procedures

First week, standing height (GMCS-IV model, China) and body composition (Inbody 720, Korea) were measured after overnight fasting (> 8 h) in the morning.

Second week, a treadmill stepwise incremental cardiopulmonary exercise test was performed to evaluate maximal oxygen uptake (VO_2max_) using indirect calorimetric measurement system (Cosmed Quark, Rome, Italy), and before the exercise test, subjects were instructed to wear a respiratory face mask in a sitting position to continuously record heart rate (Cosmed, Wireless HR Monitor) and oxygen uptake (Cosmed Quark) for 30 min, taking the middle 10 min of data and calculating the mean value as resting heart rate and resting oxygen uptake.

Third and last week, all subjects took part in two acute exercise sessions (HIIT and MICT) on a treadmill in a randomized order, and between the two exercise sessions, there was a one week wash-out period. EPOC and substrate metabolism were monitored by indirect calorimetry (Cosmed Quark) for 30 min after each exercise. Standard gas and metabolic calibration of the test system was performed before every formal test.

### VO_2max_ test

All subjects avoided any intense physical activity for 48 h prior to the test. They were also asked to abstain from caffeine and alcohol for 24 h. The Bruce treadmill protocol (Supplementary Table S1) was performed to objectively evaluate cardiorespiratory fitness. The speed and grade started at 2.7 km/h and 10%, respectively, and were increased every 3 min until the participant developed volitional fatigue. The test would terminate when the participant reached the following criteria (at least three)^19,20^: (1) with workload rising, VO_2_ was no longer rising or rose less than 2 ml/kg/min; (2) maximal heart rate reached age-predicted maximum heart rate: 220-age; (3) respiratory exchange ratio (RER) > 1.15; (4) rating of perceived exertion (RPE) ≥ 18. Oxygen uptake output was based on breath-by-breath analysis. The maximum value of the average oxygen uptake over a continuous 30-s interval was recorded as the VO_2max._

### Exercise protocols

Both HIIT and MICT were performed on a treadmill, and all participants wore a respiratory face mask during exercise interventions to achieve equal energy expenditure between HIIT and MICT. According to ACSM’s exercise prescription for weight loss, the minimum energy expenditure daily was ~ 300 kcal, therefore, this study set a goal of ~ 300 kcal of energy consumption during exercises. The exercise protocols were: (1) HIIT: 3 min bouts at 90% VO_2max_ with 2 min of recovery at 25% VO_2max_; (2) MICT: 60% VO_2max_ continuous training. All participants were instructed to avoid vigorous physical activity for 48 h and refrain from caffeine and alcohol for 24 h prior to each exercise session. Also, they were encouraged to keep dietary records for 24 h before each exercise intervention and try to maintain a consistent diet. All interventions were performed in the Human Movement Science Laboratory at Beijing Sport University. The testing room was well ventilated, and the temperature and relative humidity were 20–22 °C and 50%, respectively.

### Definition of relevant indicators of EPOC

#### Duration of EPOC

The duration during the recovery period when oxygen uptake returned to baseline period.

#### Total EPOC

Total oxygen consumption in the EPOC period subtracted the oxygen uptake in the baseline period. The duration of EPOC refers to the time after exercise when the oxygen uptake returned to the baseline level. When the oxygen uptake reached the baseline, we will continue to monitor for another 10 min to further determine.

#### Production rate of EPOC

Total EPOC/Duration of EPOC.

### Calculations of substrate metabolism

We selected subjects’ VO_2_ and VCO_2_ for 1 min, and accumulated them to obtain the total amount of VO_2_ and VCO_2_ during a certain time period to calculate substrate metabolism. According to the method proposed previously^21^ to calculate the substrate metabolism.

$${\text{Carbohydrate oxidation rate }}\left( {{\text{mg}}/{\text{kg}}/{\text{min}}} \right) \, = { 4}.{585}0{\text{ VCO}}_{{2}} \left( {{\text{ml}}/{\text{kg}}/{\text{min}}} \right) \, - { 3}.{\text{2255 VO}}_{{2}} \left( {{\text{ml}}/{\text{kg}}/{\text{min}}} \right),$$

$${\text{Lipid oxidation rate }}\left( {{\text{mg}}/{\text{kg}}/{\text{min}}} \right) \, = { 1}.{\text{6946 VO}}_{{2}} \left( {{\text{ml}}/{\text{min}}/{\text{kg}}} \right) \, - { 1}.{7}0{\text{12 VCO}}_{{2}} \left( {{\text{ml}}/{\text{kg}}/{\text{min}}} \right),$$

$${\text{Lipid energy output }}\left( {{\text{cal}}/{\text{kg}}/{\text{min}}} \right) \, = {\text{ lipid oxidation rate }}\left( {{\text{mg}}/{\text{kg}}/{\text{min}}} \right) \, \times { 9},$$

$${\text{Total energy output }}\left( {{\text{cal}}/{\text{kg}}/{\text{min}}} \right) \, = {\text{ lipid oxidation rate }}\left( {{\text{mg}}/{\text{kg}}/{\text{min}}} \right) \, \times { 9 } + {\text{ carbohydrate oxidation rate }}\left( {{\text{mg}}/{\text{kg}}/{\text{min}}} \right) \times {4},$$

$${\text{Percentage of energy from lipid }} = {\text{ Lipid energy output}}/{\text{Total energy output}}.$$

### Statistical analysis

Sample size was estimated by GPower (Version 3.1.9.2). A priori calculation was selected, power was 0.8, effect size was 0.8^22,23^, significance level α was 0.05, and 25% of the dropout rate was considered. Therefore, a total sample size of 20 subjects was set for recruitment. All data were expressed as mean ± SD. Normality of data was tested using the Shapiro–Wilk test. Student’s paired T test was used to compare the differences between HIIT session and MICT session. Statistical significance was *p* < 0.05, and *p* < 0.01 was considered to be a very significant statistical difference. All statistical analyses were performed by SPSS 25.0 (SPSS Inc., Chicago, IL, USA).

## Results

### EE during and after HIIT and MICT (Table 2)

**Table 2:** EPOC and EE between interventions.

	HIIT (n = 21)	MICT (n = 21)
Planned EE (kcal)	300	300
Actual EE (kcal)	303.32 ± 34.69	298.32 ± 19.76
Exercise duration (min)	29.50 ± 3.04**	37.60 ± 4.56
EE of EPOC (kcal)	66.20 ± 14.36*	53.91 ± 12.63
Total EE (kcal)	369.53 ± 40.26*	346.23 ± 25.32
EPOC (ml)	4343.17 ± 1723.03**	3049.78 ± 1217.93
EPOC duration (min)	28.73 ± 4.17	26.43 ± 4.24
EPOC/FFM (ml/kg)	67.86 ± 25.43**	48.79 ± 20.82
EPOC/BM (ml/kg)	48.68 ± 18.40**	35.18 ± 15.25
Production rate of EPOC (ml/min)	155.76 ± 43.66**	125.41 ± 53.58

Results are presented as mean ± SD.

*EE* energy expenditure, *EPOC* excess post-exercise oxygen consumption, *FFM* fat free mass, *BM* body mass, *HIIT* High-intensity interval training, *MICT* moderate-intensity continuous training.

*Significant difference between HIIT and MICT, *p* < 0.05.

**Indicate *p* < 0.01.

EE during exercise was no difference between HIIT (303.32 ± 34.69 kcal) and MICT (298.32 ± 19.76 kcal, *p* = 0.240), which indicated a matched EE between exercise protocols.

Exercise duration consuming ~ 300 kcal during HIIT (29.50 ± 3.04 min) was significantly shorter than during MICT (37.60 ± 4.56 min, *p* = 0.000). EE of EPOC after HIIT (66.20 ± 14.36 kcal) was higher than after MICT (53.91 ± 12.63 kcal, *p* = 0.045). Total EE during entire HIIT (369.53 ± 40.26 kcal) was higher than during entire MICT (346.23 ± 25.32 kcal, *p* = 0.043). That is, HIIT could achieve equal EE in a shorter time compared to MICT, and meanwhile, the total EE generated by HIIT is also higher than MICT.

### EPOC after HIIT and MICT

The absolute EPOC after HIIT (4343.17 ± 1723.03 ml) was significantly higher than after MICT (3049.78 ± 1217.93 ml, *p* = 0.002). Although the duration of EPOC after HIIT (28.73 ± 4.17 min) was slightly longer than after MICT (26.43 ± 4.24 min), there was no statistical difference between the two exercise interventions (*p* > 0.05). Meanwhile, EPOC production rate after HIIT (155.76 ± 43.66 ml/min) was also higher than after MICT (125.41 ± 53.58 ml/min, *p* = 0.000) (Table 2).

In this study, EPOC was monitored for 30 min, when divided into every 10 min. As shown below (Table 3), EPOC during 0–10 min after HIIT (2649.48 ± 351.42 ml) was significantly higher than after MICT (1856.73 ± 725.84 ml, *p* = 0.034), whereas no differences were found during 10–20 min and 20–30 min after HIIT and MICT (*P* > 0.05). It could be seen from the percentage of EPOC in different time periods that EPOC was mainly produced in the 10 min after HIIT and MICT.

**Table 3:** EPOC and EPOC percentage in different time periods.

	HIIT (n = 21)	MICT (n = 21)
0–10 min EPOC (ml)	2649.48 ± 351.42*	1856.73 ± 725.84
EPOC percentage (%)	69.35 ± 19.21	63.79 ± 30.13
10–20 min EPOC (ml)	892.73 ± 702.30	735.29 ± 519.06
EPOC percentage (%)	18.64 ± 8.48*	10.40 ± 7.50
20–30 min EPOC (ml)	645.29 ± 639.81	784.95 ± 554.86
EPOC percentage (%)	12.01 ± 11.39	18.60 ± 15.83

Results are presented as mean ± SD.

*EPOC* excess post-exercise oxygen consumption, *HIIT* high-intensity interval training, *MICT* moderate-intensity continuous training.

*Significant difference between HIIT and MICT, *p* < 0.05.

### Substrate metabolism during HIIT and MICT (Table 4)

**Table 4:** Substrate metabolism between interventions.

	HIIT (n = 21)	MICT (n = 21)
During exercise		
Lipid oxidation rate (mg/kg/min)	1.92 ± 1.18	2.19 ± 0.96
Lipid energy output (cal/kg/min)	17.25 ± 10.65	19.72 ± 8.69
Carbohydrate oxidation rate (mg/kg/min)	26.13 ± 6.76**	17.12 ± 5.54
Carbohydrate energy output (cal/kg/min)	104.53 ± 27.03**	68.49 ± 22.16
Percentage of energy from lipid (%)	15.07 ± 10.51**	23.09 ± 10.71
After exercise		
Lipid oxidation rate (mg/kg/min)	1.01 ± 0.43**	0.76 ± 0.46
Lipid energy output (cal/kg/min)	9.08 ± 3.84**	6.82 ± 4.16
Carbohydrate oxidation rate (mg/kg/min)	3.70 ± 1.18	3.72 ± 0.94
Carbohydrate energy output (cal/kg/min)	14.82 ± 4.71	14.88 ± 3.78
Percentage of energy from lipid (%)	37.94 ± 14.21*	30.09 ± 13.54

Results are presented as mean ± SD.

*HIIT* high-intensity interval training, *MICT* moderate-intensity continuous training.

*Significant difference between HIIT and MICT, *p* < 0.05.

**Indicate *p* < 0.01.

The lipid oxidation rate and lipid energy output were higher during MICT (2.19 mg/kg/min and 19.72 ± 8.69 cal/kg/min) than during HIIT (1.92 ± 1.18 mg/kg/min and 17.25 ± 10.65 cal/kg/min), whereas no significant differences were found (*p* > 0.05). Nevertheless, carbohydrate oxidation rate and carbohydrate energy output were both higher during HIIT (26.13 ± 6.76 mg/kg/min and 104.53 ± 27.03 cal/kg/min) than during MICT (17.12 ± 5.54 mg/kg/min and 68.49 ± 22.16 cal/kg/min, *p* = 0.000). And the percentage of energy from lipid was higher during MICT (23.09 ± 10.71%) than during HIIT (15.07 ± 10.51%, *p* = 0.000).

### Substrate metabolism after HIIT and MICT

The lipid oxidation rate and lipid energy output were very significantly higher after HIIT (1.01 ± 0.43 mg/kg/min and 9.08 ± 3.84 cal/kg/min) than after MICT (0.76 ± 0.46 mg/kg/min and 6.82 ± 4.16 cal/kg/min, *p* = 0.000). Carbohydrate oxidation rate and carbohydrate energy output did not differ between the two exercise sessions (*p* = 0.960). The percentage of energy from lipid was also higher after HIIT (37.94 ± 14.21%) than after MICT (30.09 ± 13.54%, *p* = 0.020) (Table 4).

As well, the time period of substrate metabolism after exercise was divided into 10 min. Results suggested that, over time, during the EPOC period, carbohydrate oxidation rate was gradually decreased, on the contrary, lipid oxidation rate and the percentage of energy from lipid were gradually increased. In addition, lipid oxidation rate was significantly higher during 10–30 min after HIIT than after MICT (Table 5).

**Table 5:** Substrate metabolism in different time periods.

		Carbohydrate oxidation rate (mg/kg/min)	Lipid oxidation rate (mg/kg/min)	EE (cal/kg/min)	Percentage of energy from lipid (%)
0–10 min	HIIT	5.54 ± 0.77**	0.90 ± 0.40	30.27 ± 4.03**	26.30 ± 10.49
	MICT	4.44 ± 0.46	0.85 ± 0.39	25.43 ± 3.81	29.09 ± 9.98
10–20 min	HIIT	3.06 ± 1.11	1.58 ± 0.70*	26.41 ± 8.75*	52.76 ± 13.93
	MICT	2.82 ± 0.89	1.03 ± 0.48	20.58 ± 4.65	43.97 ± 16.56
20–30 min	HIIT	1.71 ± 1.20*	1.68 ± 0.53**	21.93 ± 7.57	69.62 ± 14.46**
	MICT	2.77 ± 0.91	1.06 ± 0.37	20.63 ± 4.18	46.15 ± 14.65

Results are presented as mean ± SD.

*EE* energy expenditure, *HIIT* high-intensity interval training, *MICT* moderate-intensity continuous training.

*Significant difference between HIIT and MICT, *p* < 0.05.

**Indicate *p* < 0.01.

### Total substrate metabolism during and after HIIT and MICT (entire HIIT and entire MICT) (Table 6)

**Table 6:** Total substrate metabolism variables.

	HIIT (n = 21)	MICT (n = 21)
Total lipid oxidation rate (mg/kg/min)	1.46 ± 0.73	1.55 ± 0.68
Total lipid energy output (cal/kg/min)	13.17 ± 6.57	13.94 ± 6.12
Total carbohydrate oxidation rate (mg/kg/min)	14.72 ± 3.38**	11.06 ± 2.82
Total carbohydrate energy output (cal/kg/min)	58.86 ± 13.53**	44.24 ± 11.29
Total percentage of energy from lipid (%)	18.89 ± 10.44*	24.29 ± 10.39

Results are presented as mean ± SD.

*HIIT* high-intensity interval training, *MICT* moderate-intensity continuous training.

*Significant difference between HIIT and MICT, p < 0.05.

**Indicate *p* < 0.01.

Total lipid oxidation rate and lipid energy output were slightly higher during entire MICT than during entire HIIT, whereas no differences were seen (*p* > 0.05). Total carbohydrate oxidation rate and carbohydrate energy output were significantly higher during entire HIIT (14.72 ± 3.38 mg/kg/min and 58.86 ± 13.53 cal/kg/min) than during entire MICT (11.06 ± 2.82 mg/kg/min and 44.24 ± 11.29 cal/kg/min, *p* = 0.000). Total percentage of energy from lipid was higher during entire MICT (24.29 ± 10.39%) than during entire HIIT (18.89 ± 10.44%, *P* = 0.048).

## Discussion

### Effects of EE matched HIIT and MICT on energy consumption and EPOC after exercise

This study showed that HIIT resulted in greater EE and EPOC than EE matched (~ 300 kcal) MICT in 30 min after exercise. Due to the equal EE during the two exercise sessions, the total EE was also significantly higher during entire HIIT than during entire MICT. Moreover, exercise duration was significantly shorter during HIIT than during MICT, that is, HIIT could result in greater energy consumption and EPOC in a shorter time. Therefore, HIIT has the potential to become a more time-efficient training protocol for fat loss, but it depends on the HIIT protocols and other contributing factors (e.g. energy intake). And this can be beneficial for people with obesity who lack of time to exercise. These results could be relevant to the higher exercise intensity during HIIT. Studies have shown that exercise intensity and duration are the critical impact factors on EPOC. Six untrained healthy men were recruited in a research to perform aerobic exercise on a cycle ergometer, results suggested that, when exercise intensity remained constant, the magnitude of EPOC increased with the increase in exercise duration^24^. Another research showed greater EPOC was produced with the increase of exercise intensity when EE matched training was performed^25^. Lv^26^ recruited sixteen untrained healthy young females with obesity who performed cycling (~ 200 kJ), results showed EPOC and energy consumption of EPOC were both higher in the 90% VO_2max_ and 120% VO_2max_ HIIT groups than in the 60% VO_2_max MICT group. In our study, exercise duration was shorter during HIIT than during MICT, whereas the magnitude of EPOC and EE of EPOC were higher after HIIT than after MICT. Meanwhile, notably, the duration of EPOC was slightly longer after HIIT than after MICT, although no statistical difference was found. These results potentially suggested that higher exercise intensity might be more important than longer exercise duration in producing EPOC. And this was in line with previous studies, which suggest exercise intensity played a more critical role than exercise duration in producing EPOC after exercise^27–30^. Furthermore, an interval training pattern could be more favorable for producing EPOC than continuous training. A study^31^ found that EPOC after interval running was much higher than after continuous running. It was also showed^32^ that the duration and magnitude of EPOC after interval cycling were significantly higher than after continuous cycling. However, the EPOC response was comparable when comparing different interval training protocols, such as HIIT and sprint interval training (SIT)^29^, even if higher intensity was used in SIT. Thus, it can be speculated that there is an optimal inflection point to induce the maximum EPOC response. However, a review^29^ showed that SIT, in contrast to HIIT, produced a larger EPOC compared to MICT. Since SIT was not used in our study, we were unable to determine the difference in its effectiveness compared to HIIT. Given that the exercise bout time and recovery time of HIIT in this study was 3 min and 2 min, different exercise protocols remain to be investigated in the future.

EPOC is also affected by endogenous hormones such as catecholamine, thyroxine, and adrenocortical hormone, which are potential impact factors for energy consumption^33^. Study showed that the concentration of testosterone was increased after both moderate intensity and high intensity training, whereas the concentration was higher in the high intensity group^34^. In our study, the greater EPOC after HIIT might be associated with the high exercise intensity during HIIT, which causes a higher concentration of hormones after exercise.

With respect to the different time periods producing EPOC, it was suggested that EPOC was mainly produced in 10 min after exercise, with a statistical difference between HIIT and MICT. This was probably because the human body was still at a high metabolic level immediately after exercise, resulting in relatively high oxygen uptake and energy consumption. Although there was a significant difference in EPOC percentage (%) between the 10 and 20 min, there was no statistical difference in their absolute values between 10 and 20 min. As for EPOC, its absolute value may be more significant than the relative value.

### Effects of EE matched HIIT and MICT on substrate metabolism during and after exercise

In this study, carbohydrate oxidation rate was clearly higher during HIIT compared to MICT. Lipid oxidation rate was slightly higher during MICT compared to HIIT, meanwhile, the percentage of energy from lipid was higher during MICT than during HIIT. This was directly attributed to the high intensity of exercise during HIIT. With the increase in exercise intensity, the energy supply rate was expected to increase, resulting in a shift from lipid metabolism to carbohydrate metabolism, and accordingly, the efficiency of carbohydrate oxidation was improved^35^.

During the EPOC period, lipid oxidation rate and the percentage of energy from lipid were both higher after HIIT compared to MICT, with no difference in carbohydrate oxidation between the two exercise sessions, which indicated a higher efficiency of lipid metabolism. More glycogen resynthesis was required after HIIT might explain this phenomenon. Since a greater level of glycogen depletion occurred during HIIT compared with MICT, lipid oxidation was increased to sustain energy demand after HIIT^36,37^. Moreover, catecholamine-induced lipolysis could be a potential impact factor linked to lipid utilization after exercise. As mentioned before, the concentration of relevant hormones was higher after HIIT compared to MICT. Still, this might be associated with changes in the activity of lipid metabolism-related enzymes^38^. However, through comparison of the availability and oxidation of free fatty acids, research suggested that lipolysis during EPOC after HIIT was not superior to MICT^39^. Hence, more detailed studies are expected to focus on the biological mechanisms of lipid oxidation during the EPOC period.

As well, our study specifically examined the time course of substrate metabolism during EPOC. It was suggested that carbohydrate oxidation was gradually decreased, and a very significant difference was found in the first 10 min after HIIT compared with MICT. On the contrary, lipid oxidation and the percentage of energy from lipid were gradually increased. This could be related to the natural recovery process of the human body after exercise. In the shorter time immediately after exercise, fast energy supply is still needed. With the recovery of metabolism, a shift from fast to relative slow energy supply occurs, resulting in the transition from carbohydrate to lipid energy supply. Given that metabolic rates are highest in the first 10 min after exercise, it can be speculated that if an exercise protocol could produce as much EPOC as possible in the first 10 min, then the total magnitude and duration of EOPC, as well as the total amount of substrate metabolism, would increase. Consequently, future research might be needed to explore the effects of different exercise protocols on shorter acute EPOC.

Considering total substrate metabolism, total carbohydrate oxidation rate and carbohydrate energy output were higher during the entire HIIT compared to MICT. No difference was seen with regard to total lipid oxidation between HIIT and MICT, whereas the total percentage of energy from lipid was clearly higher during the entire MICT compared to HIIT. This was due to the significantly higher total EE during the entire HIIT compared to MICT.

From the perspective of the whole process of energy metabolism and substrate metabolism, HIIT is superior to MICT for lipid metabolism. For people with obesity, HIIT might boost their enthusiasm and save them time.

Strength of this study was that objective measurements were used to monitor the energy and substrate metabolism in different exercise periods with more detail.

However, this study also has some limitations. Firstly, the EE of EPOC between HIIT and MICT is not that huge. Given that this is an acute trial, it is possible that multiple interventions may add up to differences in clinical importance, but further experimental evidence is needed. Secondly, we only measured the acute EPOC response. And due to the contribution of anaerobic conditions, there may be biases in the calculation of EE and substrate metabolism during the HIIT execution process^40^. Thirdly, we only encouraged participants to maintain a consistent diet as much as possible and kept in touch with them to receive feedbacks, but did not use objective measurement tools. Lastly, it was also noteworthy that EPOC was affected by a combination of multiple factors, even if EE was matched. In this study, we mainly controlled sex, age and body fat, but we did not investigate hormone levels or any related mechanisms. Therefore, results should be considered with caution.

## Conclusion

In summary, An acute session of HIIT results in greater magnitude of EPOC and total EE, as well as higher percentage of energy from lipid during EPOC than EE matched (~ 300 kcal) MICT in male college students with obesity. Future researches are needed to further compare the effects of various types of HIIT programs, including SIT, on EPOC and substrate metabolism.

### Supplementary Information

Supplementary Table S1.

## Data Availability

The datasets used and analyzed during the current study are available from the corresponding author on reasonable request.
