# Supplementary material for: Acute interval running induces greater excess post-exercise oxygen consumption and lipid oxidation than isocaloric continuous running in men with obesity
Source: Sci Rep. 2024 Apr 22;14:9178. doi: 10.1038/s41598-024-59893-9 (PMC11035584; doi:10.1038/s41598-024-59893-9)
Supplement: Supplementary file 1 — Supplementary Table S1. [file 41598_2024_59893_MOESM1_ESM.pdf]

Title: Interval Running Results in Greater Excess Post-exercise Oxygen Consumption and Lipid Oxidation Than Energy Expenditure Matched Continuous Running in Male College Students With Obesity

Authors: Lang Jiang,[1,2,#] Yihong Zhang,[1,3,#] Zhengzhen Wang,[1] Yan Wang [1,*]

Affiliations: [1]School of Sports Medicine and Rehabilitation, Beijing Sport University, Beijing, China; [2]School of Public Service Management, Chongqing Vocational College of Transportation, Chongqing, China; [3]School of Physical Education, Hunan University of Science and Engineering, Yongzhou, Hunan, China

#These authors contributed equally to this work.

*Corresponding author.

Email address and contact details of corresponding author: 1991@bsu.edu.cn; Yan Wang; School of Sports Medicine and Rehabilitation, Beijing Sport University, Beijing, China.

Supplementary Table S1 Bruce protocol

Stage	Speed (km/h)	Grade (%)	Time (min)	METs	Total time (min)
1	2.7	10	3	4	3
2	4.0	12	3	7	6
3	5.5	14	3	9	9
4	6.8	18	3	13	12
5	8.0	18	3	16	15
6	8.9	20	3	19	18
7	9.7	22	3	22	21
